# Experiences of Using a Self-management Mobile App Among Individuals With Heart Failure: Qualitative Study

**DOI:** 10.2196/28139

**Published:** 2021-08-09

**Authors:** Myra Schmaderer, Jennifer N Miller, Elizabeth Mollard

**Affiliations:** 1 College of Nursing University of Nebraska Medical Center Lincoln, NE United States

**Keywords:** mHealth, eHealth, mobile applications, patient experiences, patient perceptions, self-management, self-care, heart failure, congestive heart failure, heart decompensation

## Abstract

**Background:**

Interventions that focus on the self-management of heart failure are vital to promoting health in patients with heart failure. Mobile health (mHealth) apps are becoming more integrated into practice to promote self-management strategies for chronic diseases, optimize care delivery, and reduce health disparities.

**Objective:**

The purpose of this study was to explore the experience of using a self-management mHealth intervention in individuals with heart failure to inform a future mHealth intervention study.

**Methods:**

This study used a qualitative descriptive design. Participants were enrolled in the intervention groups of a larger parent study using a mobile app related to self-management of heart failure. The purposive, convenient, criterion-based sample for this qualitative analysis comprised 10 patients who responded to phone calls and were willing to be interviewed. Inclusion criteria for the parent study were adults who were hospitalized at Nebraska Medical Center with a primary diagnosis and an episode of acute decompensated heart failure; discharged to home without services such as home health care; had access to a mobile phone; and were able to speak, hear, and understand English.

**Results:**

Study participants were middle-aged (mean age 55.8, SD 12 years; range 36-73 years). They had completed a mean of 13.5 (SD 2.2) years (range 11-17 years) of education. Of the 10 participants, 6 (60%) were male. Half of them (5/10, 50%) were New York Heart Association Classification Class III patients and the other half were Class IV patients. The intervention revealed four self-management themes, including (1) I didn’t realize, and now I know; (2) It feels good to focus on my health; (3) I am the leader of my health care team; and (4) My health is improving.

**Conclusions:**

Participants who used a self-management mHealth app intervention for heart failure reported an overall positive experience. Their statements were organized into four major themes. The education provided during the study increased self-awareness and promoted self-management of their heart failure. The mHealth app supported patient empowerment, resulting in better heart failure management and improved quality of life. Participants advocated for themselves by becoming the leader of their health, especially when communicating with their health care team. Finally, the mHealth app was used by the participants as a self-management tool to assist in symptom management and improve their overall health. Future research should study symptom evaluation, medication tracking, and possibly serve as a health provider communication platform to empower individuals to be leaders in their chronic disease management.

## Introduction

The prevalence of heart failure in adult Americans continues to increase with over 6 million diagnosed with heart failure [1]. Heart failure continues to be one of the leading causes of mortality and morbidity [2]. Self-management interventions have improved patient outcomes, such as individual’s knowledge, quality of life, and hospitalizations due to heart failure [3]. However, less is known about patients' experiences with self-management mobile health (mHealth) interventions to assist in the management of chronic heart failure.

The study of self-management techniques in chronic disease management has led to the research of patient activation and the role that an individual’s knowledge, skills, abilities, and willingness to manage chronic health conditions play in multimorbid disease processes. There is growing evidence that supports patient activation to guide interventions and assist in implementing strategies to activate individuals for self-management [4,5]. Previous studies have linked patient activation with positive health outcomes, such as decreased health care utilization [6,7], reducing symptoms [8,9], and improving quality of life [10].

Interventions that focus on the nonpharmacologic management of heart failure are vital to promoting chronic disease management in patients with heart failure. Heart failure practice guidelines recommend self-management through daily symptom and weight monitoring [11]. However, slight changes in health status can be challenging to identify. The complex nature of heart failure makes early symptom intervention vital to reducing health care utilization, hospital readmissions, and improving quality of life. Furthermore, empowering individuals to focus on their health and developing self-management routines has been shown to reduce heart failure disease burden.

Mobile phones and other portable technologies are increasingly more affordable. mHealth apps are becoming more integrated into practice to promote chronic disease self-management strategies [12], optimize care delivery, and provide potential health benefits with use [13]. Studies have shown that mHealth apps engage individuals in their health care and increase empowerment [14]. mHealth interventions have shown to promote chronic disease self-management in persons with heart failure [15,16] and patients with other chronic diseases [13,17-20].

A recent review found that mHealth apps focusing on heart failure self-management are cost-effective solutions to symptom monitoring and promoting engagement [21]. The study participants using mHealth apps showed an improvement in their quality of life, medication adherence, and reduced readmissions [21]. In other studies, participants have found the apps to be more convenient for self-management of fluid intake than traditional means because of smartphone portability and ease of data input [16]. Despite the positive outcomes of using mHealth apps among individuals with heart failure, there remain challenges to their use and uptake. Barriers to the use of mHealth apps include the lack of patient’s integration of technology in everyday life [16] and difficulties in using mobile apps [15]. In the elderly population, health problems such as cognitive changes related to aging, disability, and lack of confidence are reasons for not using digital technology [22,23]. Further research is needed to evaluate patient experiences with apps, and the benefits gained as a result.

A recent review identified few high-quality commercially available mHealth apps for managing heart failure and a paucity of peer-reviewed literature assessing the experiences, functionality, and efficacy of apps [12]. However, with more interventions using mHealth as a medium for intervention delivery, its acceptability and usability have been reported [16,24,25]. Although many heart failure apps are currently available, a systematic search [26] found that several apps need further redesign or development to engage users in self-management behaviors. It is important to measure patient experiences and perceived benefits of mHealth apps to ensure uptake and usability of future apps. The purpose of this qualitative study was to further understand and explore the experience of using a self-management mHealth app among patients with heart failure to inform a future planned mHealth intervention study.

## Methods

### Study Design

This study used a qualitative descriptive design. Qualitative descriptive research creates a close description of participants’ subjective experience [27,28]. Participants were recruited from the intervention groups of a larger parent study using a mobile app related to heart failure self-management [29]. The parent study was a three-group repeated measures randomized controlled pilot study. Institutional review board approval was obtained at a major academic institution, and all participants provided written informed consent for both the larger parent study and this qualitative study.

### Intervention Description

**Figure 1:**
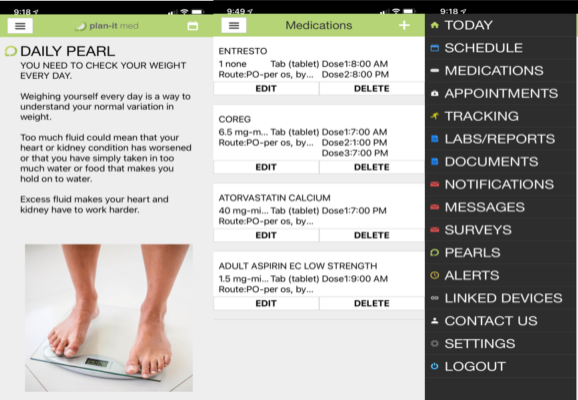
Screenshots of the mHealth app used in this study.

A brief description of the intervention will be provided in this manuscript, as a full description of the sample and study is under review in another paper [29]. The 12-week intervention promoted self-management using mHealth as a platform. Participants in all three groups (ie, enhanced usual care, mHealth, and mHealth Plus) received an mHealth app (Play-It Health) customized for the study. The app was downloaded to a mobile device (phone or tablet) with a Bluetooth incorporated weighing scale. Screenshots of the app are presented in Figure 1.

The enhanced usual care group received the app to report medications and body weights without any reminders. The mHealth group received the app and weighing scale with reminders daily to weigh in and answer educational tips (ie, “pearls”) related to heart failure. *Pearls* were evidence-based and promoted self-management behaviors for chronic disease. Additionally, reminders were sent to individuals prior to scheduled medications. The mHealth Plus group received the aforementioned components plus virtual visits with a cardiac nurse practitioner and community health worker over 8 weeks to promote self-management, symptom management, and decision-making. Virtual visits were not evaluated in this qualitative study. The intervention's theoretical underpinnings were based on Hibbard’s conceptualization of patient activation (ie, knowledge, skill, and confidence) [30].

### Sample

For this qualitative study, participants were enrolled from the intervention arms (mobile app) of the parent study. This purposive, convenient, criterion-based sample selected for this qualitative study consisted of the first 10 patients who responded to phone calls requesting their feedback on their experience with the mHealth app.

Inclusion criteria for the parent study were adults who were hospitalized at Nebraska Medical Center with a primary diagnosis and an episode of acute decompensated heart failure; discharged to home without services such as home health care; had access to a mobile phone or iPad; and were able to speak, hear, and understand English. Participants were excluded if they had documented dementia or a life expectancy of fewer than 6 months.

### Procedures

One researcher interviewed all 10 participants individually in approximately 30- to 60-minute-long qualitative interviews. The interview method followed a semistructured interview guide that allowed for both formal and informal interaction between the interviewer and participant, guided by participant response.

### Analysis

We conducted a qualitative analysis of the transcribed interview transcripts by using thematic content analysis techniques as described by Miles and Huberman [31], organized using NVivo qualitative data analysis software (version 12, 2018; QSR International Pty Ltd.). Our analysis started with individual open coding by two reviewers (including EM who had conducted the interviews), comparing and contrasting, recoding after collaborative discussion, and finally clustering of relevant codes into common themes. Themes were then discussed and reviewed with an additional peer reviewer principal investigator, MS). Saturation was determined when information obtained from participants became redundant, and no further thematic observations could be determined [32,33], Saturation was determined after analyzing 7 participants’ data, but because of previously scheduled interviews, 3 additional participants were included in the sample. 

## Results

### Overview

Demographic and clinical characteristics are represented in Table 1. Participants of this subsample were generally middle aged (mean age 55.8, SD 12 years), in the age range of 36 to 73 years. The mean years of education was 13.5 (SD 2.2) with a range 11 to 17 years. Of the 10 participants, 6 (60%) were male; 4 (40%) were White, 4 (40%) were Black, and 1 (10%) was American Indian, whereas 1 self-reported more than one race. Half of the participants (5/10, 50%) were uninsured, 3 (30%) had private insurance, and 2 (20%) were enrolled in Medicare. Regarding acuity, half were New York Heart Association Classification Class III patients, and the other half were Class IV patients (5/10, 50% each). According to American College of Cardiology Foundation/American Heart Association Heart Failure Staging System, most (7/10, 70%) were Stage C patients, whereas 2 (20%) were Stage B patients and 1 (10%) was a Stage D patient. The majority were patients with heart failure (9/10, 90%), with reduced ejection fraction whereas the remaining 1 (10%) had preserved ejection fraction. This subsample had demographic characteristics typical of the parent study.

**Table 1:** Demographic and clinical characteristics of the study sample (N=10).

Characteristic			Value
Age in years, mean (SD); range			55.8 (12); 36-73
Educational level in years, mean (SD); range			13.5 (2.2); 11-17
**Gender, n (%)**			
	Female	4 (40)	
	Male	6 (60)	
**Race, n (%)**			
	Caucasian	4 (40)	
	Black	4 (40)	
	American Indian or Pacific Islander	1 (10)	
	More than one race	1 (10)	
**Employment, n (%)**			
	Yes	3 (30)	
	No	5 (50)	
	Retired	2 (20)	
**Marital status, n (%)**			
	Married or cohabitating	4 (40)	
	Single or separated	5 (50)	
	Divorced	1 (10)	
**Insurance, n (%)**			
	Uninsured	5 (50)	
	Medicare	2 (20)	
	Private insurance	3 (30)	
**New York Heart Association classification, n (%)**			
	Class III	5 (50)	
	Class IV	5 (50)	
**American College of Cardiology Foundation/American Heart Association Heart Failure Stage classification**			
	Stage B	2 (20)	
	Stage C	7 (70)	
	Stage D	1 (10)	
**Ejection fraction, n (%)**			
	<50%	9 (90)	
	≥50%	1(10)	

### Study Themes

**Figure 2:**
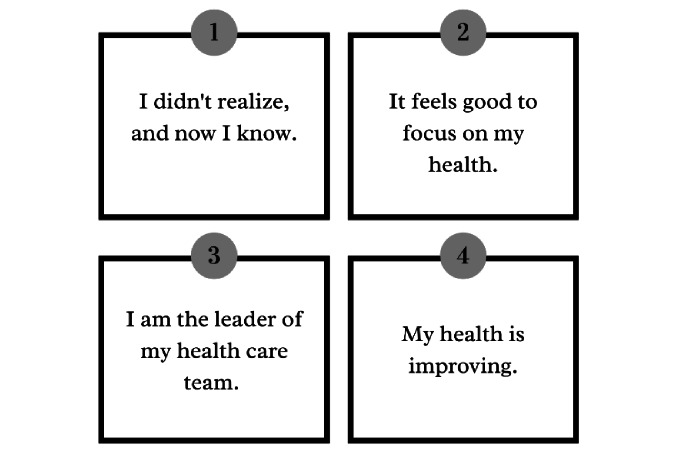
Self-management themes emerged from participants’ experience with the mHealth app.

Four final themes emerged, characterizing the experiences of individuals using the mHealth app. The intervention provoked four self-management themes, focusing on their chronic illness: (1) I didn’t realize, and now I know; (2) It feels good to focus on my health; (3) I am the leader of my health care team; and (4) My health is improving (Figure 2).

#### I Didn’t Realize, and Now I Know

Participants noted that by using the self-management intervention, they became in tune with their health. Through frequent monitoring of medication administration, body weight, and vital signs, participants began to realize that changes to their health were occurring daily. The app inspired self-monitoring—a critical element of self-management. One younger participant (aged 39 years) noted that he was inspired to begin monitoring because of the app, “It made me pay more attention to myself and want to check myself again, ’cause I don’t want the same thing happen again, you know.” [Participant 29]. Another participant noted that by using the app to monitor all elements of her health, including self-managing behaviors combined with the specific education from *pearls*, she felt a new awareness and comfort.

With that medication reminder and then weighing myself every day to see if I was retaining water, it helped me a lot because I was really scared at first. But now that I’ve taken my meds the same time and took everything more seriously and learned everything that I got - everything that I know about heart failure and heart disease, I learned it from that app because of those little questionnaires you got.

Participant 78

Another participant spoke about how the regular self-monitoring could be related back to his behaviors and tell him more information about what may trigger changes to his health:

It’s been interesting for one reason. I seem to have a weight gain when I go goose hunting...So, I think that’s an interesting part of this, is that I can go back and see well, what was I three or four or five days and ago...So, it’s very interesting to me how I can peak and then I had come back down during the week and then I might go up again and, so.

Participant 71

#### It Feels Good to Focus on My Health

After participants began the regular practice of monitoring their health status through the mobile app, they began to feel hopeful and empowered to be focusing on their health.

I love the scale. I love weighing myself every morning. That’s a good thing. I don’t ever want to go through that build-up of fluids in my body again. That was kind of miserable. You know and I get on the scale every morning.

Participant 76

Many participants noted the transformation from feeling afraid and powerless after receiving their diagnosis or being hospitalized to the change toward empowerment and feeling more in charge once they began using the app:

I was actually thinking I was going to die. But, now that I know a little bit more and how I can improve my health and my heart, I have a lot of hope and when the doctor told me that, you know, I’m good, I’m okay now, you know, like with everything I’m doing, I’m doing everything right.

Participant 78

#### I Am the Leader of My Health Care Team

Participants noted that their experience with their health care providers also improved, as they were more empowered with knowledge and information about their health. This allowed them to speak up more with their health care providers and provide essential health information. One participant noted:

It really helped enabling me to make me feel like I was actually more a part of the process of the treatment.

Participant 18

Another participant noted that having information about her body weight improved her experience at her appointment and allowed her to weigh carefully at home:

I have a lot of doctor’s appointments and, you know, I have several issues and when I go to my appointments, I’m in a wheelchair and it’s kind of painful to stand up. So, I can show them in my phone a record of my actual weight for the morning...I like being able to, like, go into the doctor’s appointment, show exactly what my weight was and it really made me aware of keeping up with my weight, my ups and downs and how much I’ve gained.

Participant 39

Participants also noted that the organization of the app improved the interactions with their health care team, likely improving the care they received. One participant noted how the app had improved her health care interaction as opposed to pen and paper:

Sometimes I take off to the doctor and I forget it [weight records on paper]. Well now I can click on my phone and go to that app that you know from you guys and boom they’re all listed right there on my phone so I can show them to my doctor. You know how the nurse always checks your pills before you go in, and I just show them that and they see everything that I’ve got on.

Participant 76

#### My Health is Improving

Many individuals with chronic illness, specifically heart failure or multimorbid conditions do not believe that their health can improve. Although participants began to monitor their health, feel good about managing it, and interact in clearer ways with their health care team, they also began to notice that these behaviors were also correlated with an improvement in their health. One participant noted:

I was able to, like, you know, better myself. My heart was only working at a 25% when I first started the app and now...my heart is improved to a 45% strength then.

Participant 78

Other participants noted that while their health was improving, it became even clearer to themselves that the self-management behaviors they had learned through the mobile app were critical to their health maintenance and improvement going forward.

It really did help and my condition got really this bad because it just really got this bad as a result of me being neglectful and it was a good thing it was caught in time because it took a while for me to really come to the conclusion of what was actually going on and so I’m really grateful for that.

Participant 18

## Discussion

### Principal Findings

Participant experience using the mHealth interventions were overall positive and enhanced their self-management of heart failure symptoms. Statements received by participants were organized into four major themes: First, education provided during the study increased self-awareness and promoted self-management through a better understanding of heart failure. Second, the mHealth app supported participant empowerment, resulting in better self-management of their condition of heart failure and, in turn, increasing quality of life. Third, the participants felt activated to take responsibility for their health by advocating for themselves and communicating with their health care team. Finally, participants felt the mHealth app provided them with a tool to self-manage their symptoms and, therefore, improve their health. These specific themes support previous research findings that mHealth apps can be an empowering and engaging platform for participants to better manage chronic illnesses [14,21].

Studies have shown that education focusing on symptom awareness enhances self-management, thus enhancing the ability to recognize subtle health changes. It is important for future mHealth interventions to incorporate a chronic illness education component to emphasize disease management and monitoring of symptoms. Moreover, technological literacy, patient activation, and cognition should also be considered [34-36]. In relationship to the theme *I didn’t realize, now I know*, the mHealth intervention incorporated an educational component within the app consisting of a daily educational tip (*pearl*) related to heart failure. Education is a common component of mHealth and self-management research in mobile health [21,24]. This theme deepened the understanding of how education can be incorporated into apps and interventions. The app offered a small daily *pearl* related to heart failure, followed by a question for the users to answer about the content shared on the given day. Participants received positive encouragement when the question was answered correctly. Educational content tailored to the participant’s knowledge level enhances self-management and the ability to recognize subtle health changes [35]. The mHealth app in this study offered a different and unique strategy to present educational content that may be less overwhelming to the participants than large amounts of content presented at once. Effective strategies to deliver education within such apps warrants further study.

The theme *It feels good to focus on my health* supports the empowerment of patients and likely enhances their ability to manage their heart failure symptoms. Empowering and activating patients to manage their chronic disease can increase the quality of life and reduce health care utilization [18,37]. Likewise, persons with low activation levels may be at greater risk of poor health outcomes and unplanned hospital readmissions in persons with chronic disease [7,38]. A study completed in rural heart failure patients found a decreased understanding of their health made the self-management of chronic illness sometimes impossible because of decreased knowledge around illness-related symptoms [34]. Consistent with a recent review on mobile apps for individuals with chronic diseases [14], this study identified that participants were engaged and empowered when using the app. An increase in the activation levels may encourage health-related behaviors, such as increasing physical activity, dietary behaviors, and medication adherence [39,40], as well as support closer monitoring of their disease symptoms [18].

The theme *I am the leader of my health care team* was supported by participant reports that the mHealth app empowered them to lead conversations with their health care team. Participants were able to advocate for themselves by communicating with their providers. Having health information readily available on their phone was convenient and less cumbersome than having paper and pencil records. mHealth apps should be used as a complement to health care with a provider [14], as they may offer a direct communication pathway via technology or can be accessed on a mobile device during face-to-face visits. Apps can promote the education needed for individuals to know how to manage their disease process at home and when to communicate with their health care team for more support [13,18,41]. These mHealth interventions aim to test ways to reduce the impact of chronic illness by preventing exacerbations, empowerment self-management skills, and encouraging communication with the health care team when appropriate.

Some participants reported the theme *My health is improving* in the realization that self-monitoring with the app correlated with improving their health and managing their heart failure. Findings from this study were consistent with other reported research [17], identifying that apps are often a useful platform to record daily biomarkers such as weights. The ability to visualize the trend of stable or decreasing weight offers a strategy to see health changes. Other apps also incorporate identifying symptoms, such as breathing, daytime sleepiness, or fatigue [12,42]. Built-in medication reconciliation tools may help participants communicate with their health care team, promoting up-to-date recording keeping and accurate reporting that is consistent with other mHealth apps [12,17,19,43]. Enhancements to this app could include the ability to notify a health care provider when biomarkers or symptoms change from day-to-day, as previously reported [10].

### Limitations

Future research with a larger sample size is needed to control for potential bias. This sample was purposive and convenient. Participants were telephoned at various times of the day and the first 10 individuals were interviewed. It is important to learn from those less engaged and seek strategies to improve the app and intervention; likewise, it is imperative to know how the mHealth app benefits some individuals. It is timely to study mHealth interventions limiting face-to-face contact, considering the recent COVID-19 pandemic.

### Future Research

In the future, we plan to include participants in the development of the intervention to assess their needs and desires to enhance engagement. These results combined with the parent study’s results on usability and acceptability of the intervention will inform a larger, fully powered study.

Future research should focus on the components of mHealth interventions that improve outcomes. One possible approach is to incorporate food logs or daily symptom evaluation to further enhance the mHealth intervention. It is also essential to evaluate notification fatigue; for example, how many notifications should be sent and how often they should be sent. Research has shown that a patient-centered approach with app tailoring options allows for personalization options and will guide the frequency and timing of notification in future studies [14].

### Conclusions

The use of mHealth can enhance the promotion of self-management techniques in patients with heart failure. Enhancing patient engagement is directly affected by patients’ usability of an mHealth intervention and potential benefits from its tailored content. This study showed that participants who were interviewed about the self-management mHealth app intervention for heart failure reported an overall positive experience. The education provided during the study increased their self-awareness and promoted self-management of their heart failure. Future mHealth apps need to support patient empowerment, resulting in better heart failure management and improved quality of life. Participant activation to take responsibility for their health through advocating for themselves when communicating with their health care team is vital to long-term self-management of chronic diseases. The mHealth app we evaluated could also be used in future studies to assist in symptom management and, therefore, improve individual’s overall health. Future research will further support symptom evaluation, medication tracking, and possibly serve as a health provider communication platform to empower individuals to be leaders in their chronic disease management.

## Abbreviations

mHealth: mobile health
